# Supplementary material for: “Do we even have a voice?” Health providers’ perspective on the patient accommodation strategies in Bangladesh
Source: PLoS One. 2022 Aug 1;17(8):e0271827. doi: 10.1371/journal.pone.0271827 (PMC9342766; doi:10.1371/journal.pone.0271827)
Supplement: S1 File — (DOCX) [file pone.0271827.s001.docx]

**Annexure**

**Interview guide**

1. How important do you think it is to interact with patients in the maternity ward for effective service delivery?
2. Can you describe the facilitators of effective communication? (I mean, how do you think you can communicate the best possible ways in the health complex)
3. What are the barriers, in your opinion, to effective interaction with patients? (Can you share an experience where you felt difficulties communicating with a patient or relatives)
4. What do you feel when/if you consider that you cannot communicate/interact with patients the way you would have liked? How that affects the overall treatment or service operation (discourse management)?
5. Can you share your experience where you might have felt intimidated by the prospect of not delivering the care the patients might expect from you? How do you adjust in situations like this?
6. How did you accommodate patients when there was any difference in language and culture with patients?
7. How important for you to comfort patients emotionally and share the medical decision to facilitate decision making?
8. What are your opinions, if there any, on improving communication with patients based on the experience you have?
9. What kind of expertise do you think providers need to achieve before being involved with the patients in the real world?
10. What kind of behaviors do you expect from patients (Ideal scenario/environment where you would like to deliver your service?)
