# Supplementary material for: “Do we even have a voice?” Health providers’ perspective on the patient accommodation strategies in Bangladesh
Source: PLoS One. 2022 Aug 1;17(8):e0271827. doi: 10.1371/journal.pone.0271827 (PMC9342766; doi:10.1371/journal.pone.0271827)
Supplement: Attachment — Submitted filename: Rebuttal letter_Plos_10.02.2022.docx [file pone.0271827.s002.docx]

“Do We Even Have a Voice?” Health providers’ perspective on the patient accommodation strategies in Bangladesh

The author would like to thank the Editor and the two reviewers for their valuable comments and insights that helped to improve the manuscript. We have tried to answer all the issues mentioned by the reviewers and update some parts based on the suggestions received. Please find the details below where we have tried to clarify points one by one. Two references added to the (Introduction part, Reference number: 11, 13) reference list due to their relevance to the literature.

**Comments from reviewers and responses from the author:**

1. Discussion should be based on CAT, although results section does include integration of CAT.

**Response:** Thank you for this important comment. We have updated the discussion section, which focused more on CAT, although CAT was discussed in the middle part of the discussion section already.

2. In the methodology section, how and on what grounds the participants were selected? What inclusion and exclusion criteria was followed?

**Response:** The study used a convenience sampling technique and considered healthcare providers who were directly involved with patients and their relatives in the maternity ward in providing delivery care or other care related to maternal and childcare. The study interviewed doctors, midwives, and nurses who had a significant role in maternal health service operations. This study did not consider health providers who were not directly involved with maternity care.

3. If no saturation was achieved, then justify 10 participants for the study?

**Response:** Initially, it was planned to include more participants for this study. But due to covid-19, it was difficult to reach health providers even if they work in maternity care mostly. They were involved in other patients' care as well, which made them a very difficult group of people to reach in this unprecedented period of time. The study did encounter saturation to some extent; however, inflicting saturation might be a little far-fetched since only limited participants were available. The final number of participants was decided based on their availability, timing, and free schedule. No generalization of the findings was targeted. The study tried to deliberately include a diverse group of participants (doctors, nurses, and midwives) to explore and understand the diversity in perceptions of communication with patients and their diverse accommodation strategies.

4. Selection of hospitals was done on what grounds? Are both hospitals similar? From the methodology section it appears that 1 is secondary health institution and 1 is primary health institution. How can you justify using 2 different levels of health institutions? For study to have some uniformity, both institutions have to be of similar nature.

**Response:** The study targeted public health facilities in Bangladesh, so in that aspect, both the facilities were publicly funded and selected based on convenience of access. The study was not intended to compare the primary and secondary level of care; it was intended to assess health providers' perceptions about their interaction with patients in different levels of healthcare and how they accommodate their patients based on their level of facilities. Of course, similarities-dissimilarities were discussed, but the basis was to have a deeper understanding of common grounds.

5. How reliability and validity of the data (transcriptions) was ensured.

**Response:** Probing questions were asked to participants to prevent ambiguity or double meaning. Data curation and analysis were assisted by one research associate who also independently read and coded the data set to validate the process through multiple angles. We focused only on interview responses to limit researcher’s subjectivity. The principal researcher and associate went through all the data sets to find out any incongruity in their coding based on the initial codebook developed by the principal researcher. The process was rechecked multiple times (from field notes to transcriptions to theme development) till the generation of themes, so that discrepancies could be reconciled and resolved. The process is thoroughly discussed in the data analysis section.

6. The study has one perspective only that is service provider perspective. In my opinion if patients are also included the study would have had more strength. This is one future direction that needs to be explicitly stated in future directions.

**Response:** I agree with you completely and therefore, I already included it in the limitation section (last part of the discussion). Conclusion and policy implication is based on the current study findings. A future research is under consideration which will also include patients’ perspectives. Thank you for this great suggestion.

7. The manuscript talks about the experience of mistreatment, discrimination, and disrespect in a resource constrained setting in a developing country. This manuscript concentrates on the degree of interaction with pregnant women particularly in the context of accommodating patients and their relatives to improve communication between the parties of health care as well as patients. Manuscript also mentions of systematic limitations of health care facilities and heavy workload reported by staff. Poor attitudes and neglect from some patients and providers perception of interaction with patients therefore their adopted accommodation strategies for providing health care services were studied. This was analyzed using communication accommodation strategy (CAT). Communication accommodation theory which is one of the prominent behavioral theories of communication and the author provides a thematic analysis in table 1. Provider’s perception of patient interaction and their adopted accommodation strategies were studied. Due to systematic limitations of health care facilities and heavy workload especially nurses and midwives reported poor attitudes and neglect from some patients. The health care providers also use different approaches to accommodate patients emotional and psychological needs through verbal and nonverbal clues. The author request for prompt delivery of health care services and awareness programs to urge patients and visitors to cooperate with medical procedures, so they can collaborate with current settings as much as possible. Health care sector can also consider facilitating providers workload and maintaining a power balance and service management.

**Response:** Thank you for your comment.
