# Supplementary material for: “Do we even have a voice?” Health providers’ perspective on the patient accommodation strategies in Bangladesh
Source: PLoS One. 2022 Aug 1;17(8):e0271827. doi: 10.1371/journal.pone.0271827 (PMC9342766; doi:10.1371/journal.pone.0271827)
Supplement: Attachment — Submitted filename: Response to reviewers_2_16.06.2022.docx [file pone.0271827.s004.docx]

PONE-D-21-37993R1

“Do We Even Have a Voice?” Health providers’ perspective on the patient accommodation strategies in Bangladesh

**Reviewer #3:** The manuscript can be improved further if the comments inserted in the document are addressed/considered.

**Reviewer #4:** The authors addressed the points raised by the reviewers. However the limitations imposed by the small and not so diverse group of interviewees in the study persist in some extent.
I also suggest a final review of the text. There are some minor mistakes which should be corrected.

**Author’s response:** Thank you for your comments and suggestions which we belief have made us more focused to improve our work further.

We addressed all the comments/suggestions made by the reviewers and tried to improve the manuscript. We went through the manuscript again, according to your suggestion, to find out and correct minor mistakes, where applicable. We agree with you regarding the relatively small sample size, to some extent. We tried to make the sample as heterogenous as possible involving doctors, nurses, and midwives as participants within our limits.
